# A Cognitive-Emotional Model to Explain Message Framing Effects: Reducing Meat Consumption

**DOI:** 10.3389/fpsyg.2021.583209

**Published:** 2021-03-29

**Authors:** Valentina Carfora, Massimiliano Pastore, Patrizia Catellani

**Affiliations:** ^1^Department of Psychology, Catholic University of the Sacred Heart, Milan, Italy; ^2^Department of Developmental and Social Psychology, University of Padua, Padua, Italy

**Keywords:** framing, message, meat consumption, message elaboration, emotion, message framing

## Abstract

We tested the plausibility of a cognitive-emotional model to understand the effects of messages framed in terms of gain, non-loss, non-gain, and loss, and related to the health consequences of red/processed meat consumption. A total of 544 Italian participants reported their attitude toward reduced red/processed meat consumption and intention to eat red/processed meat (time 1 questionnaire). One week later, participants were randomly assigned to four different message conditions: (a) gain messages focused on the positive health outcomes associated with low meat consumption; (b) non-loss messages focused on the avoided negative health outcomes associated with low meat consumption; (c) non-gain messages focused on the missed positive health outcomes associated with high meat consumption; (d) loss messages focused on the negative health outcomes associated with high meat consumption (message sending). After reading the messages, participants answered a series of questions regarding their emotional and cognitive reactions to the messages, their evaluation of the messages, and again their attitude and intention toward red/processed meat consumption (time 2 questionnaire). Comparing different multivariate linear models under the Bayesian approach, we selected the model with the highest plausibility conditioned to observed data. In this model, message-induced fear influenced systematic processing, which in turn positively influenced message evaluation and attitude, leading to reduced intention to consume red/processed meat. Vice versa, message-induced anger reduced systematic processing, which in turn negatively influenced message evaluation, and led to no effect on attitude and intention. The comparison among message conditions showed that gain and non-loss messages activated integrated emotional and cognitive processing of the health recommendation, while loss and non-gain messages mainly activated emotional shortcuts toward attitude and intention. Overall, these results advance our comprehension of the effects of message framing on receivers' attitudes and intentions.

## Highlights

- Gain, non-loss, non-gain, and loss messages have differential effects on attitudes and intentions.
- Message-induced fear enhances systematic processing and positive evaluation of the message.
- Message-induced anger reduces systematic processing and positive evaluation of the message.
- Gain and non-loss messages trigger integrated emotional and cognitive processing of the message.
- Gain and non-loss messages have a positive impact on future attitude and intention.
- Loss and non-gain messages activate emotional shortcuts toward attitude and intention.

## Introduction

High consumption of red/processed meat has been recognized as connected to the risk of developing various diseases, such as cancer and type 2 diabetes (Misra et al., 2018; Bianchi et al., 2019). For this reason, health authorities have recommended eating a maximum of three servings per week (e.g., Bach-Faig et al., 2011). However, many individuals still eat too much red/processed meat (e.g., Farchi et al., 2017), due to the presence of several individual barriers, such as habits or the lack of knowledge about the nutritional value of plant-based diets (Stoll-Kleemann and Schmidt, 2017).

In the domain of communication research, many scholars have investigated how to overcome the aforementioned psychological barriers, with message interventions focused on health, environmental, and ethical issues connected to high red/processed meat consumption (from now on RPMC) (e.g., Bertolotti et al., 2016; Carfora et al., 2019a,b; Stea and Pickering, 2019; Harguess et al., 2020). Only limited research has been devoted to the effects on attitude and intentions of messages framed in terms of the valence of the expected outcomes (i.e., in terms of gain, non-loss, non-gain, or loss; Di Massimo et al., 2019; Carfora et al., 2020a). In addition, so far, no scholars have considered the cognitive and emotional processing underlying the effects of such message framing (Rothman and Baldwin, 2012). Providing more evidence on how to frame messages to reduce RPMC and analyzing the factors through which message framing influences people's responses is therefore a substantial research challenge.

Starting from the above, in the present study we investigated the effects of message framing on attitudes and intentions regarding RPMC relying on two main theoretical frameworks. The first theoretical framework is the self-regulatory framework (Higgins, 1997; Cesario et al., 2013), which makes assumptions regarding the effectiveness of emphasizing positive or negative outcomes in a persuasive message. The second theoretical framework is the revised Elaboration Likelihood Model, according to which the persuasive effect of a message is useful to deepen people's cognitive and emotional responses when receiving a message (Petty and Briñol, 2015). The integration of these theoretical frameworks helped in the understanding of how to formulate messages on reduced RPMC and why they are effective (or not) in changing attitude and intention.

### Message Framing

Across an array of research traditions, past studies have demonstrated that persuasive messages induce attitude change, which in turn lead to intention and behavior change (e.g., Ajzen, 1991; Eagly and Chaiken, 1993; Wood, 2000; Petty and Cacioppo, 2012; Petty and Briñol, 2015). Focusing on the content or the construction of the message, researchers have also shown that the persuasive effect of communication depends on how message recommendations are framed (Davis, 1995; Chong and Druckman, 2007; Spence and Pidgeon, 2010). Message framing refers to the evidence that decision-makers respond differently to different but objectively equivalent descriptions of the same issue (Kühberger, 1998, p. 150), that is messages stressing the positive or negative consequences of a behavior (e.g., Rothman et al., 2006). A positively framed message presents behavioral consequences with a positive valence. Conversely, a negatively framed message presents behavioral consequences with a negative valence. Framing the expected outcomes, however, is not limited to the basic positive vs. negative valence distinction. According to the self-regulatory framework proposed by Cesario et al. (2013), both positively and negatively framed messages can also be formulated by describing the presence or absence of pleasure or pain. This level of framing refers to the so-called *outcome sensitivities level* of message framing. According to this distinction, positively framed messages can be further diversified in messages focused on *gain*, when they describe the *presence of positive outcomes* (e.g., If you eat well, you will improve your health), or on *non-loss* when they focus on the *absence of negative outcomes* (e.g., If you eat well, you will avoid damaging your health). Likewise, negatively framed messages can be further diversified in messages focused on *loss*, when they emphasize the presence of negative outcomes (e.g., If you eat badly, you will damage your health) or *non-gain*, when they inform about the *absence of positive outcomes* (e.g., If you eat badly, you will miss the opportunity to improve your health).

Regarding the effectiveness of message framing, past studies have shown that presenting the avoidance of negative consequences can be more effective than presenting the otherwise-equivalent gain, due to the “robust psychological phenomenon” of negativity bias (Cacioppo and Gardner, 1999, p. 206), that is, the heightened impact of and sensitivity to information on negative consequences. In other words, non-loss-framed messages may tend to be more effective than gain-framed messages (for a review, see Kühberger et al., 1999). The negativity bias has been related to one of the main tenets of bib47's prospect theory (1979), i.e., loss aversion, according to which people prefer avoiding losses to acquiring equivalent gains. In consideration of the negativity bias and loss aversion, we should also expect an advantage for loss messages as compared to positively framed messages. In a meta-analysis about the relationship between message framing and message processing, O'Keefe and Jensen (2008) found that gain-framed messages (i.e., messages phrased in terms of desirable states) were more involving than loss-framed messages (i.e., messages phrased in terms of undesirable states). However, the available cases did not provide evidence concerning the distinction among loss and non-loss messages. Consistent with this prior evidence, in the case of messages focused on reducing RPMC, recent studies (Di Massimo et al., 2019; Carfora et al., 2020b) showed that loss-framed messages were the least persuasive, while non-loss-framed messages, focused on the possibility of avoiding the negative consequences related to high RPMC, were the most persuasive messages, able to involve and persuade the majority of receivers independent of their prior beliefs. One possible explanation of the lower persuasiveness of loss messages as compared to non-loss messages is that the former might be more likely to trigger strong negative emotions and, in turn, reactance (Brehm and Brehm, 1981).

Even though message framing effects have been studied extensively in communication advocating different types of health behavior (e.g., Gallagher and Updegraff, 2012; Rothman et al., 2020), most research on reducing RPMC has so far ignored the distinction among gain, non-loss, non-gain, and loss messages. Recently, Di Massimo et al. (2019) and Carfora et al. (2020b) indeed tested the effects of these four types of messages, showing not only that they differentially influence attitude and intention toward RPMC, but also that their influence varies according to receivers' baseline attitude, intention, perceived efficacy, and subjective norm. To move further into the comprehension of the factors that may underlie the different effectiveness of the four types of messages, in the present study we explored the reactions receivers have when they are exposed to these messages, in terms of systematic and heuristic processing of the messages, positive or negative emotional reactions triggered by the messages, and message evaluation. We aimed to assess the cognitive and emotional mechanisms underlying message influence on attitude and intention toward reduced RPMC, as well as possible differences in the role played by these mechanisms according to message type. Below, the expected cognitive and emotional mechanisms are discussed in detail.

### Cognitive Processes Involved in Message Evaluation

The higher or lower effectiveness of different ways of framing messages depends on how these messages are processed (e.g., Meyers-Levy and Maheswaran, 2004), and message processing can be usefully investigated referring to two classic dual-process models of persuasion: the elaboration likelihood model (Petty and Cacioppo, 1986) and the heuristic systematic model (Eagly and Chaiken, 1993). The basic premise of these models is that attitude and intention changes depend upon the likelihood that an issue or argument will be positively evaluated by the receiver. Message evaluation has a direct effect on receivers' attitude and intention toward the recommended behavior (e.g., Cauberghe et al., 2009), and this effect has also been demonstrated when the recommended behavior is the reduction of RPMC (Bertolotti et al., 2020a,b). Message evaluation can therefore be considered an important proximal determinant of the framing effect on attitude and behavior change.

Message evaluation is strongly affected by systematic or heuristic processing (Chaiken, 1980), that is, by differences in the amount of cognitive effort an individual devotes to processing and thinking about a message. Systematic processing implies cognitive effort in considering the content of a message and its relevance to a given attitude object, such as a behavior. Heuristic processing is a type of shortcut that individuals use when they are less motivated to or able to think carefully about the message. When this is the case, individuals simply rely on some non-message aspects of communication to decide whether they agree or not with the message content. In the present study, we considered receivers' self-reported systematic message processing as an important precursor of message evaluation. In doing so we referred to the work of Smerecnik et al. (2012), who developed a scale to quickly gauge whether people systematically or heuristically process message information.

### Emotional Processes Involved in Message Evaluation

Although the classic dual-process models have produced decades of convincing results, they have also been criticized for undervaluing the role of emotions during message processing and evaluation (Kitchen et al., 2014). Research focusing on framing effects has also generally focused on theories mainly designed to capture the “rational” processes of decision making, overlooking the possibility that other discrete emotions, such as fear or anger, might influence framing effects. However, several scholars have shown a clear role of affective responses while processing and evaluating a message (e.g., Gross and D'ambrosio, 2004; Dillard and Nabi, 2006; Peters et al., 2006; Kühne et al., 2015).

One of the emotions that is more likely to influence the processing and thus the evaluation of a message is fear. A long history of research has led to the general conclusion that messages inducing fear are more effective than those that do not (for a meta-analysis, see De Hoog et al., 2007), also in relation to attitude and intention change toward a variety of health-related behaviors (for a meta-analysis, see Tannenbaum et al., 2015). However, messages inducing fear have also been shown to be counterproductive, and it is still not clear under which conditions this is more likely to be the case (Popova, 2014). On the one hand, fear can attract attention to the message and directly influence information processing (Loewenstein et al., 2001). Messages evoking fear lead people to rely on systematic processing, which in turn stimulates many issue-relevant thoughts, and thus a positive message evaluation (e.g., Meijnders et al., 2001; Slater et al., 2002; Meyers-Levy and Maheswaran, 2004). On the other hand, fear can induce people to enact defensive strategies to reduce the potential emotional distress associated with the message. For example, not focusing attention on the message, or reinterpreting or disregarding its content (Witte, 1992; Ruiter et al., 2001). However, so far, no research has considered how the reactions to message-induced fear may be influenced by message framing based on the self-regulatory framework.

Another emotion that is likely to influence the processing and evaluation of a message is anger. Previous research showed that persuasive messages framed with the appraisals of certainty, control, and blame can trigger anger, and the intensity of felt anger in turn determines processing ability and subsequent behavioral intentions (Turner, 2007; Walter et al., 2019). Some studies also showed that angry people are more inclined to recur to accessible and relevant heuristics when processing information that otherwise they would process analytically (Moons and Mackie, 2007). In the case of persuasive messages, recipients are often aware of the persuasive intent of the message and may feel that the message threatens their freedom of opinion and action. This feeling activates reactance aimed at the reestablishment of the threatened freedom, leading recipients to react with anger, counterarguments, as well as attitudes and behavior that run counter to the message intent (Dillard and Shen, 2005; Rains, 2013). To the best of our knowledge, however, no research has analyzed how exposure to message framing based on the self-regulatory framework induces anger in recipients, in turn influencing message processing and evaluation. In the present study, we investigated whether this would be the case and whether anger would play a role in influencing attitude and intention change toward RPMC.

### The Present Study

Based on the above literature, in the present study we proposed and tested a composite theoretical model to understand the cognitive and emotional mechanisms activated by message exposure in the case of gain, non-loss, non-gain, and loss messages on RPMC reduction. Our starting point was the model proposed and tested by Di Massimo et al. (2019) and Carfora et al. (2020b), in which baseline attitude, baseline intention, and message evaluation were key predictors of attitude and future intention to engage in RMPC, although differently according to the same four message conditions.

In the present study, we aimed at further assessing the processes that underlie message persuasiveness and framing effects. We expected that a model considering both cognitive and emotional dimensions would best explain receivers' message evaluation, attitude, and intention after reading the messages. To test this expectation we compared the fit of different models: (a) a model in which we considered the relationships between baseline attitude and intention toward RPMC and message evaluation, and in turn, the relationships between message evaluation and receivers' attitude and intention toward RPMC after reading the messages; (b) a pure cognitive model in which we considered the possible mediating role of systematic and heuristic processing; (c) a pure emotional model in which we considered the possible mediating role of message-induced fear and message-induced anger; and (d) an integrated cognitive-emotional model in which we considered both the cognitive (systematic and heuristic processing) and the emotional dimensions (message-induced fear and anger) as predictors of message evaluation, attitude, and intention.

**Figure 1:**
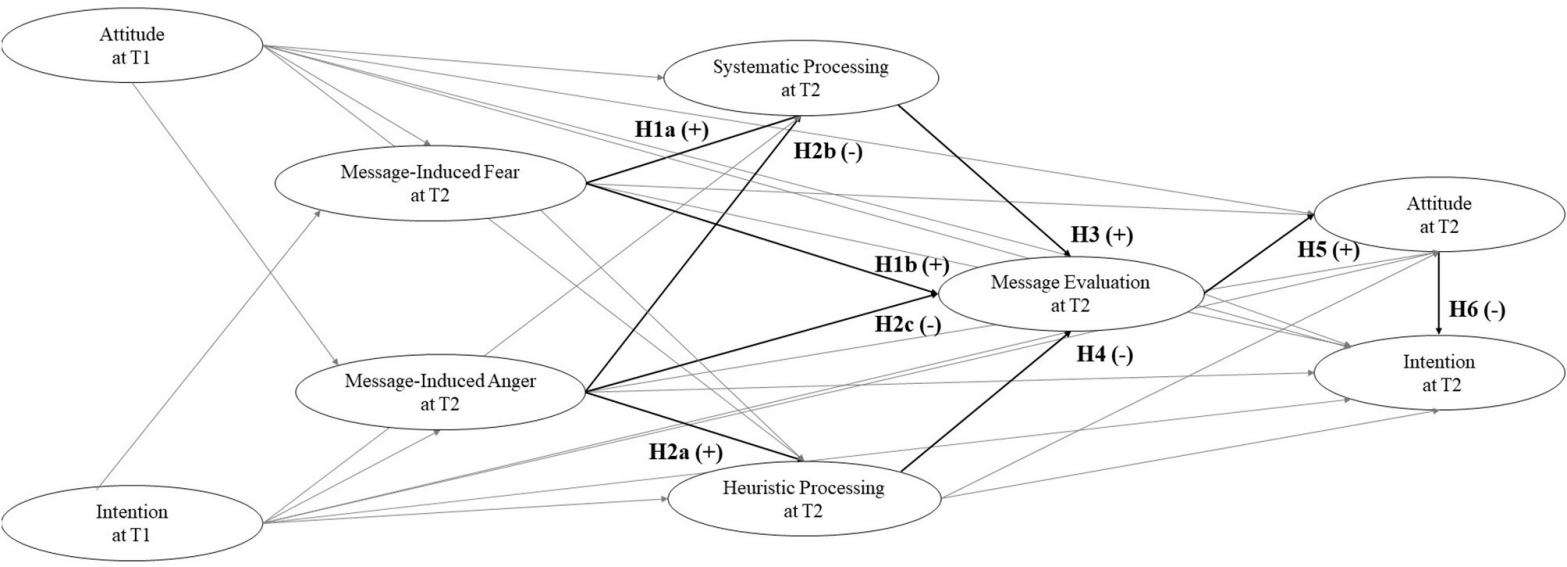
A cognitive-emotional model to explain message framing effects. Gray lines represent additional paths considered in the model.

In consideration of what was discussed in the introduction, we expected that the last and integrated model would be the one with the highest plausibility conditioned to observed data. This model is illustrated in Figure 1. Based on the literature discussed above, we tested these main hypotheses:

1. message-induced fear positively influences systematic processing (hypothesis 1a, H1a) and message evaluation (hypothesis 1b, H1b);
2. message-induced anger positively influences heuristic processing (hypothesis 2a, H2a), and negatively influences both systematic processing (hypothesis 2b, H2b) and message evaluation (hypothesis 2c, H2c);
3. systematic processing positively influences message evaluation (hypothesis 3, H3);
4. heuristic processing negatively influences message evaluation (hypothesis 4, H4);
5. a more positive evaluation of the message leads to higher attitude toward reduced RPMC (hypothesis 5, H5);
6. a more positive attitude toward reduced RPMC leads to lower intention to eat red/processed meat (hypothesis 6, H6).

In the model, possible additional direct relationships among study variables are also controlled for. They include the influence of baseline (i.e., at time 1, T1) attitude and intention on all study variables after exposure to the messages (i.e., at time 2, T2). They also include direct relationships among the hypothesized predictors of message evaluation (systematic processing, heuristic processing, message-induced fear, and message-induced anger), and attitude and intention on the other. Finally, the direct effect of message-induced fear on heuristic processing is also controlled for.

A further aim of our study was to assess whether the hypothesized relations between variables would occur in all message conditions (gain, non-loss, non-gain, loss), and if so with what intensity. Given that literature on the cognitive and emotional processes involved in the four different types of framing effects is scarce, we did not make specific hypotheses in this regard, but only a series of research questions.

How far does message-induced fear influence systematic processing (research question 1a, RQ1a) and message evaluation (research question 1b, RQ1b) in the four different message conditions?

How far does message-induced anger negatively influence systematic processing (research question 2a, RQ2a) and message evaluation in the four message conditions (research question 2b, RQ2b)?

How far does systematic processing influence message evaluation in the four different message conditions (research question 3, RQ3)?

How far does heuristic processing influence message evaluation in the four different message conditions (research question 4, RQ4)?

How far does a more positive evaluation of the message lead to greater attitude toward reduced RPMC in the four message conditions (research question 5, RQ5)?

How far does a more positive attitude toward reduced RPMC lead to lower intention to eat red/processed meat in the four message conditions (research question 6, RQ6)?

## Method

### Participants

A number of Italian citizens were invited to participate in a university study on public communication. People received an email with a link to an online questionnaire developed through the Qualtrics platform (time 1—T1). One week after the completion of the questionnaire, again through the Qualtrics platform, participants were automatically and randomly assigned to four different conditions (gain, non-loss, non-gain, and loss messages) in the ratio 1:1:1:1 and were invited to read eight messages on the health consequences of eating red/processed meat. After reading the messages, participants were required to fill in a second questionnaire (time 2—T2). The initial sample was made of *N* = 834 participants. Since the aim of the present study was to explore research questions and not to test specific hypotheses, we did not perform a power analysis to estimate the sample size (Amrhein et al., 2019). Participants who already ate <3 portions of red and processed meat per week (*N* = 96), participants who followed a specific diet (i.e., veganism, vegetarianism, or restrictive diets, *N* = 124), and participants who did not fully or accurately complete both questionnaires (*N* = 70) were then excluded. So, the final sample consisted of 544 participants, precisely 257 males and 288 females with an age ranging from 18 to 70 years (mean age = 39.97, *SD* = 14.78). In total, 9.8% of the participants had a primary level of education, 41.2% had a secondary level of education, 47.7% had a higher level of education, and the remaining 1.3% preferred not to declare. In addition, 38.9% of the participants were unmarried, 41.20% were married, 8.80% lived together, 6% were separated or divorced, 0.30% were widows, and the remaining 4.8% preferred not to declare their civil status. Participants were randomly distributed in the four message conditions as follows: gain message condition *N* = 134; non-loss message condition *N* = 134; loss message condition *N* = 141; non-gain message condition *N* = 135.

### Pre-test Measures

At the beginning of the first questionnaire participants reported their age, gender, education, and typical diet (e.g., veganism, vegetarianism or restrictive diets). Then, they read a definition of “red/processed meat consumption” (“red/processed meat is defined as mammalian meat, that is red when it is raw and dark in color when cooked. This includes beef, lamb, pork, venison, and goat, and processed meat, like beef burgers, bacon, sausages, etc. One serving is roughly the same size as a deck of cards, that is, at least two servings of vegetables per day”). After that, participants responded to a series of questions aimed at measuring their baseline attitude and intention toward RPMC.

*Attitude toward reduced RPMC* was measured using a semantic differential scale ranging from “1” to “7” (e.g., “eating little red/processed meat is… bad—good”; Carfora et al., 2017). Higher values indicated a more positive attitude toward a reduced red/processed meat consumption. Cronbach's alpha was 0.91.

*Intention toward RPMC* was assessed with three items on a seven-point Likert scale (e.g., “In the next month, how often do you intend to eat red/processed meat?”; never (1)—every day (7); Carfora et al., 2017). Higher scores indicated a greater intention to eat little red/processed meat. Cronbach's alpha was 0.97.

### Message Intervention

One week after completing the first questionnaire all participants were invited to read eight messages (~14 words each) describing the health consequences of eating red/processed meat, and formulated in prefactual terms (“if only…”; see Carfora et al., 2019a,b). Participants read different messages according to the experimental condition to which they had been randomly assigned. Participants in the *gain message condition* read messages on the positive health outcomes associated to little RPMC (e.g., “If you eat little red meat and cold cuts, you will improve the health of your stomach”). Participants in the *non-loss message condition* read messages informing about how eating little red/processed meat is connected to preventing negative health outcomes (e.g., “If you eat little red meat and cold cuts, you will avoid damaging the health of your stomach”). Participants in the *non-gain message condition* read messages emphasizing how eating excessive red/processed meat is related to missing out on positive health consequences (e.g., “If you eat a lot of red meat and cold cuts, you will miss the chance to improve the health of your stomach”). Finally, participants in the *loss message condition* read messages about the negative health outcomes of eating too much red/processed meat (e.g., “If you eat too much red meat and cold cuts, you will damage the health of your stomach”). The full list of messages is reported in Appendix 1.

### Post-test Measures

After reading the messages, participants were administered a questionnaire aimed at measuring the dimensions described below.

*Systematic processing* was measured with five items, asking participants to state how deeply they had processed the information presented in the messages (e.g., “I tried to think about the importance of the information for my daily life”; adapted from Smerecnik et al., 2012). Answers were given on a 7-point Likert scale, from (1) “strongly disagree” to (7) “strongly agree.” Higher values indicated a deeper processing of the messages. Cronbach's alpha was 0.87.

*Heuristic processing* was measured with five items, asking participants to state how superficially they processed the information presented in the messages (e.g., “While reading the messages I did not think about the arguments presented”; adapted from Smerecnik et al., 2012). Answers were given on a 7-point Likert scale, from (1) “strongly disagree” to (7) “strongly agree.” Higher values indicated higher heuristic processing of the messages. Cronbach's alpha was 0.70.

*Message-induced fear* was measured with six items pertaining to the degree to which reading messages had made participants feel fearful (e.g., “To what extent when reading these messages did you feel scared?”; adapted from Brown and Smith, 2007). Answers were given on a 7-point Likert scale, from (1) “not at all” to (7) “completely.” Higher values indicated a higher participant's fear after reading the messages. Cronbach's alpha was 0.91.

*Message-induced anger* was measured with three items related to how irritated the receivers felt after reading the messages (e.g., “To what extent when reading these messages did you feel irritated?”; adapted from Brown and Smith, 2007). Answers were given on a 7-point Likert scale, from (1) “not at all” to (7) “completely.” Higher values indicated a higher participant's anger after reading the messages. Cronbach's alpha was 0.80.

*Message evaluation* was measured with six items asking participants to state how involved they had been in the messages (e.g., “The message was very interesting”; adapted from Godinho et al., 2016). Answers were given on a 7-point Likert scale, from (1) “strongly disagree” to (7) “strongly agree.” Higher values indicated a higher participant's positive evaluation of the messages. Cronbach's alpha was 0.90.

Finally, we measured receivers' attitude toward reduced RPMC and intention to eat red/processed meat after the message exposure, with the same scale used at time 1. Cronbach's alpha was 0.99 for attitude and 0.78 for intention.

### Data Analysis

We adopted a fully Bayesian approach (Kruschke and Liddell, 2017) and all analyses were performed with the R software and programming language (R Development Core Team, 2016), with the rstan (Carpenter et al., 2017; Stan Development Team, 2018) and blavaan (Merkle and Rosseel, 2015) packages. Following a model selection approach (Burnham and Anderson, 2003; Fox, 2015), we first compared a series of multivariate models, to test our hypotheses and to assess which model would have the best plausibility conditioned to the observed data. Following the rationale exposed in the introduction and summarized in “the present study” section above, we considered and compared the following models.

- Model 0 (M0), a null model assuming no co-variances amongst the observed variables.
- Model 1 (M1), a baseline model estimating the association between attitude and intention at T1 and message evaluation, attitude, and intention at T2.
- Model 2 (M2), testing the associations of M1 plus those related to systematic and heuristic processing.
- Model 3 (M3), testing the associations of M1 plus those related to message-induced fear and message-induced anger.
- Model 4 (M4), testing the associations among all considered variables (Figure 1).

In each model, parameters were simultaneously estimated by using a multigroup approach considering the four different message conditions (gain, non-loss, non-gain, loss).

To select the best model, we considered the leave-one-out cross-validation information criterion (LOOIC) (Vehtari et al., 2017), where lower values suggest a better fit to the data, and Akaike weights, which represent an estimate of the probability that the model will make the best prediction in new data conditional upon the set of models considered (Burnham and Anderson, 2003; Wagenmakers and Farrell, 2004). Each model was fitted using the Bayesian Markov Chain Monte Carlo estimation method based on 4,000 iterations in four chains considering 8,000 post-warmup draws. Convergence was assessed by examining the potential scale reduction factor (PSRF) (Gelman and Rubin, 1992). By adopting a model comparison approach, we were able to estimate which model would provide the best explanation of the data.

After identifying the best model, we investigated the relationships among variables in each message condition. To do so, we analyzed parameter posterior distributions and summarized these distributions using posterior means and 90% highest posterior density intervals (HPDI) (Tiao and Box, 1973; Kruschke, 2011). Differently from confidence intervals in the frequentist approach, HPDI provides a direct representation of the most credible values of the estimated parameter (coefficient regression in the current study) after accounting for prior believes. A 90% HPDI represents the narrowest interval containing 90% of posterior samples. When HPDI does not include 0 (or it only contains a small proportion of values that are close to zero), it is reasonable to conclude that 0 is not a credible value and therefore an effect and/or an association can be reasonably supported.

## Results

### Model Selection

Table 1 reports the goodness of fit indices of the four models tested and the null model. Consistent with our expectation, in Model 2 the addition of the systematic and heuristic processing of the message to the basic Model 1 increased the model capacity to predict participants' attitude and intention toward RPMC. Similarly, in Model 3 the addition of message-induced fear and anger to the basic Model 1 increased the goodness of fit, and the increment was higher when compared to the one from Model 1 to Model 2. Finally, the cognitive-emotional model including all considered variables (i.e., Model 4) was the best model to predict participants' attitude and intention after message exposure. This model had the lowest LOOIC and the highest model weight and offered support to our six research hypotheses (H1—H6) regarding the relations among message-induced fear and anger, systematic processing, message evaluation, and attitude toward reduced RPMC.

**Table 1:** Model comparison results.

	**LOOIC**	**se.LOOIC**	**W**
M4	9980.91	121.93	1.00
M3	10171.51	122.01	0.00
M2	10372.05	124.94	0.00
M1	10545.82	126.38	0.00
M0	14368.72	147.68	0.00

*LOOIC, leave-one-out cross-validation information criterion (Vehtari et al., 2017); se.LOOIC, standard error; W, Akaike weight*.

### Comparison Among Message Conditions

After selecting Model 4 as the best model, we analyzed the parameter estimates of the model in the four message conditions (gain, non-loss, non-gain, loss). All parameter estimates are reported in Appendix 2. Below we will consider the predictors of all endogenous dimensions, but we will focus our comments especially on the cognitive and emotional predictors related to our six main research questions, namely, how message-induced fear predicted systematic processing (H1a; see section Systematic Processing) and message evaluation (H1b; see section Message Evaluation), how message-induced anger predicted heuristic processing (H2a; see section Heuristic Processing), systematic processing (H2b; see section Systematic Processing), and message evaluation (H2c; see section Message Evaluation), how systematic processing predicted message evaluation (H3; see section Message Evaluation), how heuristic processing predicted message evaluation (H4; see section Message Evaluation), how message evaluation was related to attitude toward reduced RPMC (H5; see section Attitude Toward Reduced RPMC at T2), and finally how attitude toward reduced RPMC was related to intention to eat red/processed meat (H6; see section Intention to Eat Red/Processed Meat at T2).

#### Message-Induced Fear

To interpret the effects of baseline attitude toward red/processed meat consumption and intention to eat red/processed meat on message-induced fear, we used the posterior distribution of regression coefficients (90% HDPI intervals are included in square brackets). The comparison among message conditions showed that in the gain message condition participants were less scared by the message when they had a high baseline positive attitude toward reducing RPMC (β = −0.07; [−0.14; −0.001]). In the other message conditions message-induced fear was instead independent of baseline attitude. In no message condition was message-induced fear predicted by baseline intention to eat red/processed meat.

#### Message-Induced Anger

Participants in the gain, non-loss, and loss conditions felt less message-induced anger when they had a more positive attitude toward reduced RPMC at time 1 (gain message condition: β = −0.17; [−0.25; −0.10]; non-loss message condition: β = −0.13; [−0.22; −0.03]; loss message condition: β = −0.13; [−0.23; −0.03]). This was not the case for participants in the non-gain message condition, who were irritated regardless of their attitude at T1. Finally, in all message conditions no significant relationship between receivers' intention at T1 and message-induced anger emerged. Thus, the feeling of anger after message exposure was independent of baseline intention to eat red/processed meat.

#### Systematic Processing

In all conditions, higher message-induced fear stimulated systematic processing and higher message-induced anger inhibited it, albeit with a different degree. Message-induced fear led to systematic processing in all message conditions (H1a), albeit more in the gain (β = 0.80 [0.17; 0.46]) and in the loss message conditions (β = 0.91; [0.61; 1.20]) than in the non-loss (β = 0.61; [0.33; 0.90]) and non-gain (β = 0.67; [0.37; 0.98]) conditions (RQ1a). Conversely, message-induced anger inhibited systematic processing in all conditions (H2b), albeit more in the gain (β = −0.92; [−1.22; −0.63]) and in the loss conditions (β = −0.79; [−1.05; −0.72]) than in the non-loss (β = −0.64; [−0.91; −0.38]) and the non-gain (β = −0.45; [−0.74; −0.15]) conditions (RQ2a).

**Figure 2:**
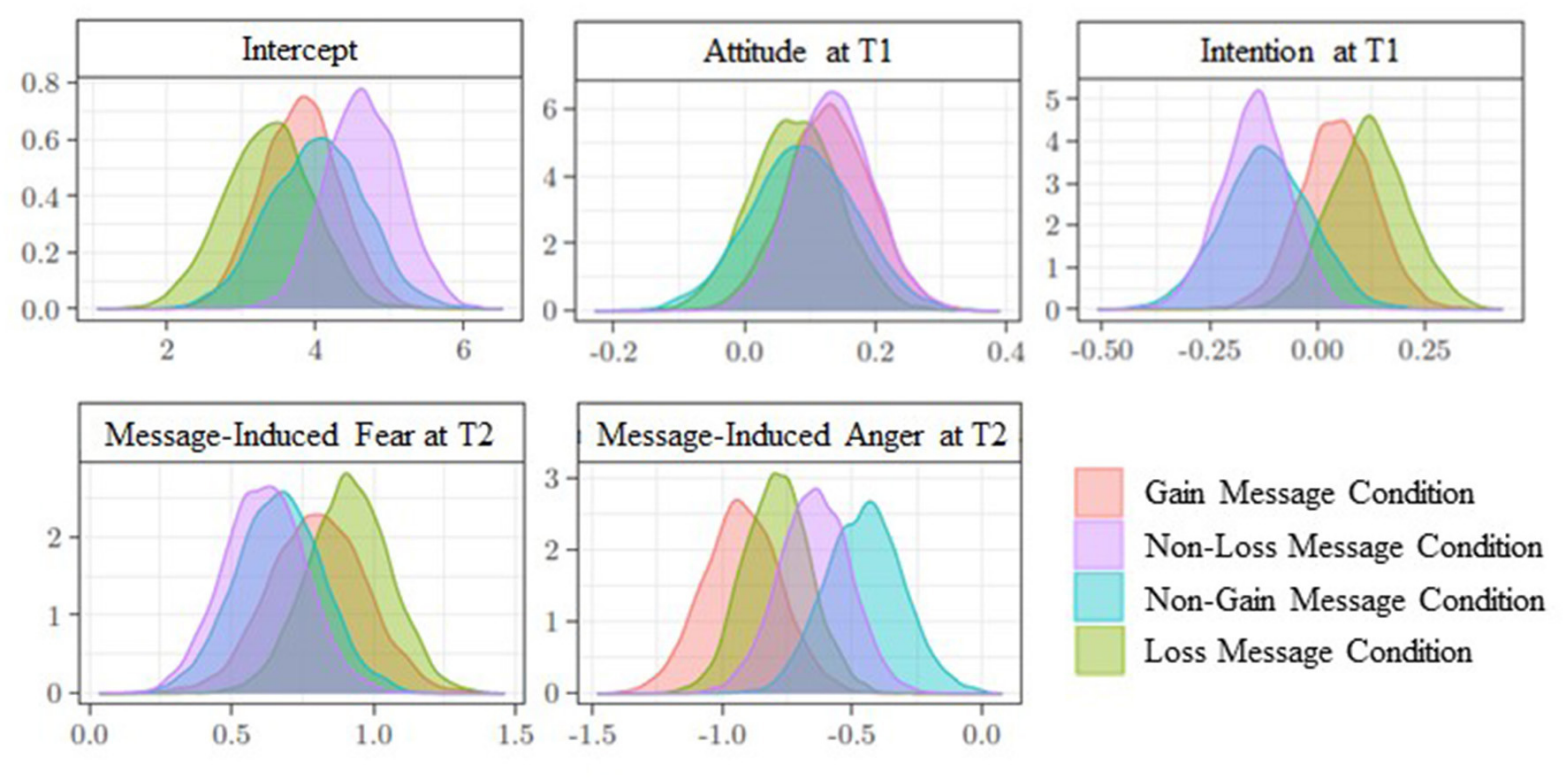
Systematic processing at time 2. Posterior distributions of the parameters associated with predictors, according to message condition.

As to the other predictors of systematic processing, exposure to gain and non-loss messages induced more systematic processing of the message when receivers had a positive attitude toward reduced RPMC (gain message condition: β = −0.13; [0.002; 0.26]; non-loss message condition: β = 0.14; [0.06; 0.02]). This was not the case in the other two conditions (non-gain message condition: β = 0.09; [−0.07; 0.24]; loss message condition: β = 0.08; [−0.05; 0.21]). Finally, intention at T1 did not influence the degree to which participants processed the message systematically. For a representation of the posterior distributions of all coefficient regressions associated with the predictors of systematic processing in each message condition, see Figure 2. In each panel of the figure, the pink curve refers to data of the gain message condition, the purple curve is related to data of the non-loss message condition, the light blue curve refers to data of the non-gain message condition, and the green curve represents data of the loss message condition.

#### Heuristic Processing

In the gain, non-loss, and loss message conditions the more participants felt anger, the more they processed the message heuristically (H2a; gain message condition: β = 0.45; [0.15; 0.17]; non-loss message condition: β = 0.45; [0.17; 0.73]; loss message condition: β = 0.23; [0.02; 0.45]). Conversely, in the non-gain message condition receivers engaged in heuristic processing regardless of their experienced anger. In all conditions the other predictors (attitude and intention at T1 and message-induced fear) did not influence participants' heuristic processing.

#### Message Evaluation

**Figure 3:**
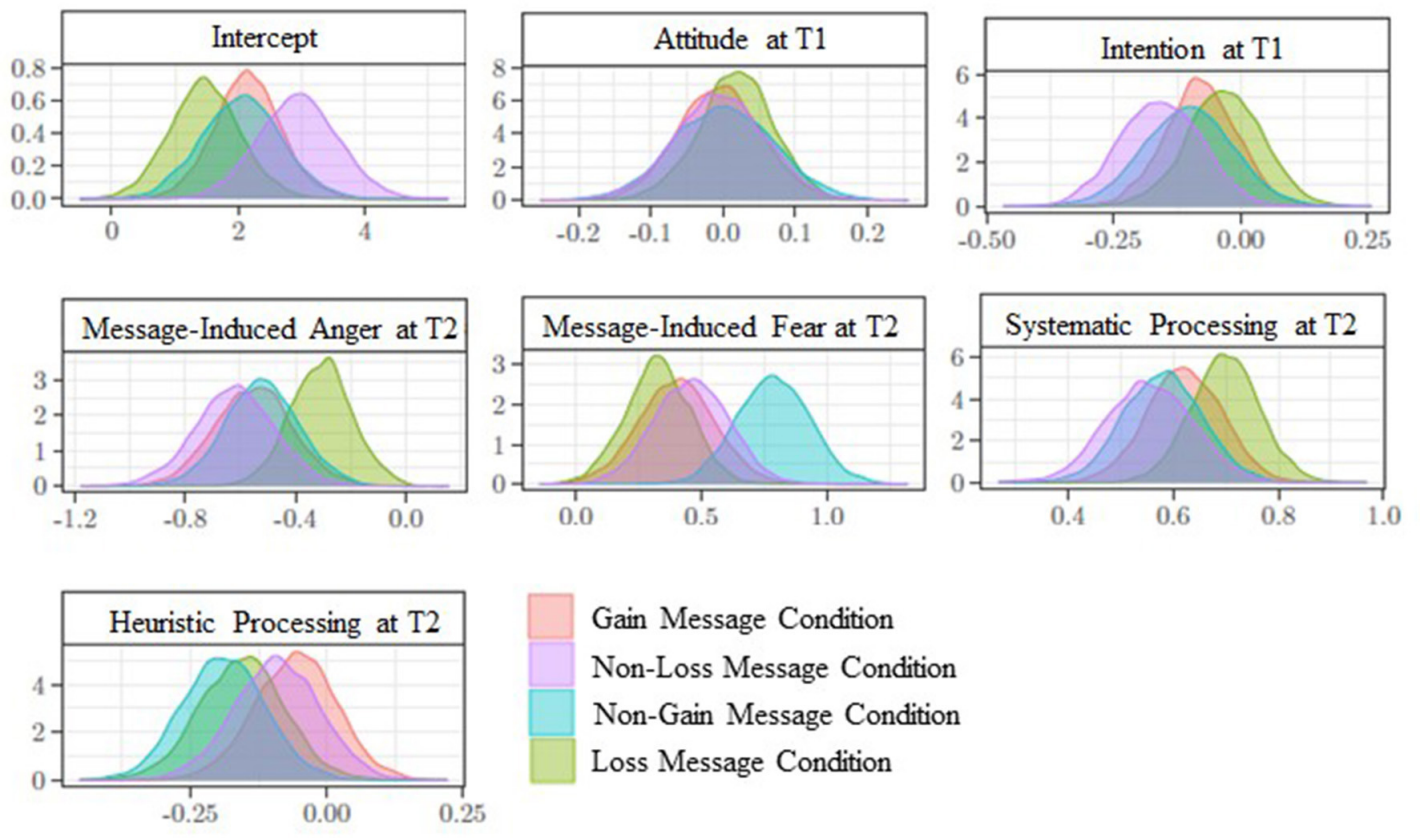
Message evaluation at time 2. Posterior distributions of the parameters associated with the predictors, according to message condition.

Moving on to the predictors of message evaluation, message-induced fear positively predicted message evaluation in all conditions (H1b), and especially in the non-gain condition (β = 0.79; [0.51; 1.07]) (RQ1b). Conversely, message-induced anger negatively predicted message evaluation in all message conditions (H2c), and this was especially the case for the non-loss condition (β = −0.62; [−0.91; −0.35]) (RQ2b). Moreover, in all message conditions systematic processing positively predicted message evaluation (H3), albeit with a different degree (RQ3; gain message condition: β = 0.62; [0.48; 0.76]; non-loss message condition: β = 0.55; [0.40; 0.71]; non-gain message condition: β = 0.58; [0.44; 0.72]; loss message condition: β = 0.70; [0.57; 0.83]). Heuristic processing negatively predicted message evaluation only in the non-gain (β = −0.19; [−0.34; −0.04]) and loss (β = −0.15; [−0.31; −0.00]) message conditions (H4; RQ4). In all conditions the other predictors (attitude and intention at T1) did not (or only marginally) predict receivers' message evaluation. Posterior distributions of each coefficient regression associated with the predictors of message evaluation in all conditions are shown in Figure 3. Table 2 shows the regression estimates of message evaluation.

**Table 2:** Posterior regression estimates of message evaluation and attitude at T2 in the four message conditions.

	**Message evaluation at T2**				**Attitude at T2**			
**Message condition**	**Gain**	**Non-loss**	**Non-gain**	**Loss**	**Gain**	**Non-loss**	**Non-gain**	**Loss**
Attitude at T1	−0.01	−0.00	0.00	0.02	0.09	0.43^*^	0.17^*^	0.29^*^
Intention at T1	−0.08	−0.16	−0.10	−0.03	−0.30^*^	−0.15	−0.36^*^	−0.14
Message–induced fear at T2	0.40^*^	0.46^*^	0.79^*^	0.32^*^	−0.31	−0.03	0.33^*^	−0.21
Message–induced anger at T2	−0.55^*^	−0.62^*^	−0.52^*^	−0.30^*^	0.16	0.00	−0.43^*^	0.00
Systematic processing at T2	0.62^*^	0.55^*^	0.58^*^	0.70^*^	0.19	−0.06	0.07	0.02
Heuristic processing at T2	−0.06	−0.09	−0.19^*^	−0.15^*^	−0.01	−0.02	0.08	−0.04
Message evaluation at T2	-	-	-	-	0.33^*^	0.34^*^	0.11	0.18

* *90% HPDI of the regression parameter does not include 0, thus the direct effect can be reasonably supported*.

#### Attitude Toward Reduced RPMC at T2

**Figure 4:**
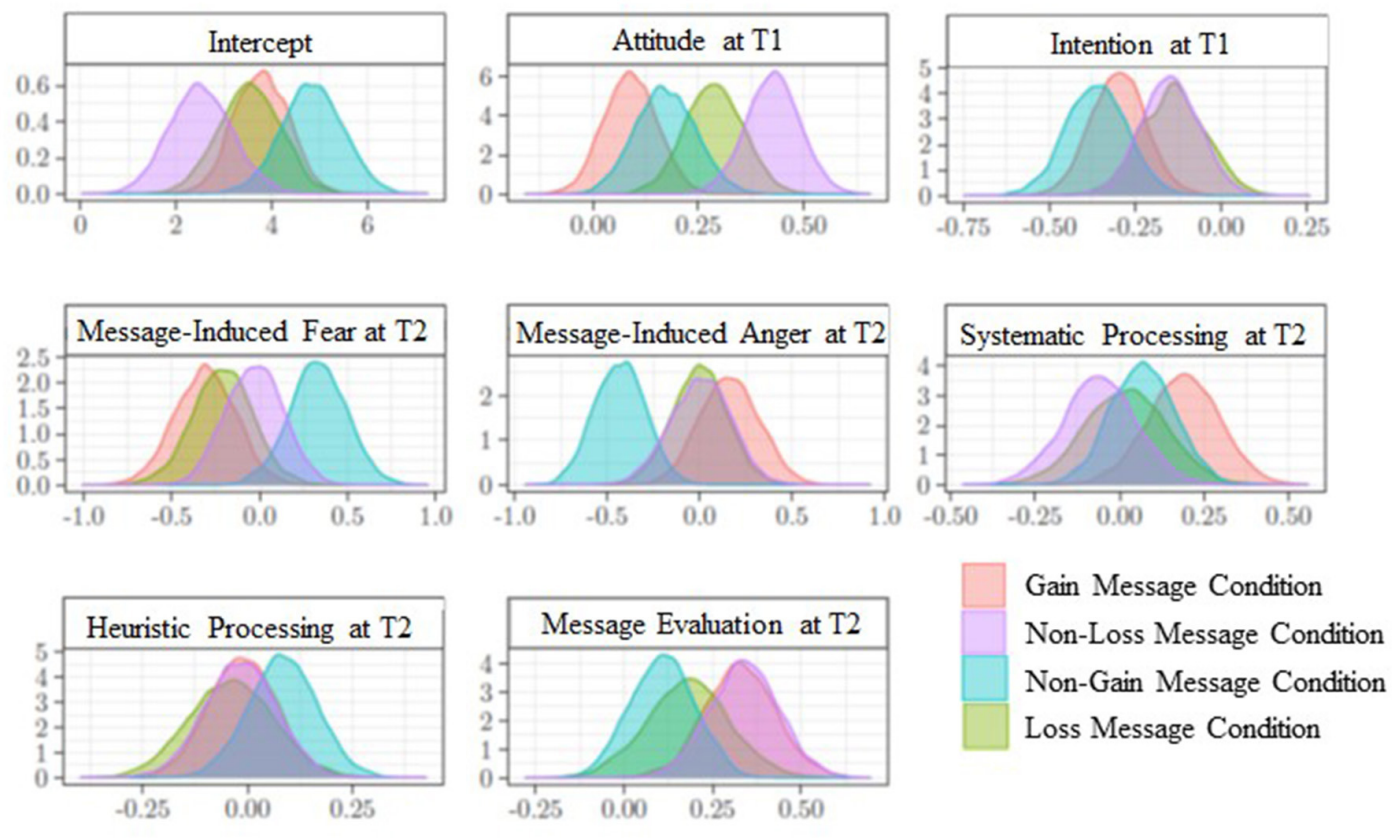
Attitude at time 2. Posterior distributions of the parameters associated with predictors, according to message condition.

Positive attitude at T2 was predicted by positive message evaluation in the case of the gain (β = 0.33; [0.13; 0.53]) and the non-loss message conditions (β = 0.34; [0.15; 0.53]), but not in the case of the non-gain (β = 0.11; [−0.07; 0.29]) and the loss (β = 0.18; [−0.05; 0.41]) conditions (H5; RQ5). In addition, only in the case of gain messages did participants show a greater positive attitude toward reduced RPMC when they also reported a lower intention to eat red/processed meat at T1 (β = −0.30; [−0.46; −0.14]). In the case of loss messages, attitude at T2 was only predicted by attitude at T1 (β = 0.29; [0.27; 0.42]). Finally, in the case of non-gain messages, attitude at T2 was not affected by message evaluation but by higher message-induced fear (β = 0.33; [0.00; 0.66]) and lower message-induced anger (β = −0.43; [−0.71; −0.15]), showing that such messages influenced attitude at T2 through an emotional processing of its content. The posterior distributions of the parameters associated with the predictors of attitude toward reduced RPMC, divided by message conditions, are shown in Figure 4. Table 2 shows the regression estimates of attitude at T2.

#### Intention to Eat Red/Processed Meat at T2

The posterior distributions of the parameters associated with the predictors of the intention to eat red/processed meat, divided by message condition, are shown in Figure 4. In all conditions, lower intention to eat red/processed meat at T2 was influenced by higher attitude toward reduced RPMC at T2 (H6 and RQ6; gain message condition: β = −0.22; [−0.34; −0.09]; non-loss message condition: β = −0.16; [−0.30; −0.02]; loss message condition: β = −0.12; [−0.23; −0.02]; non-gain message condition: β = −0.12; [−0.23; −0.003]). Again in all conditions intention at T2 was related to intention at T1 (gain message condition: β = 0.40; [0.28; 0.52]; non-loss message condition: β = 0.44; [0.30; 0.58]; loss message condition: β = 0.55; [0.43; 0.67]; non-gain message condition: β = 0.38; [0.25; 0.51]). Finally, lower intention at T2 was influenced by higher message-induced fear both in the gain [β = −0.36; [−0.61; −0.1]) and in the non-gain (β = −0.24; [−0.46; −0.02]) message conditions.

In sum, the above results fully confirmed our expectation according to which model including both cognitive (systematic and heuristic processing) and emotional (message-induced fear and anger) dimensions would be best suited to explain the effects of exposure to messages on the health consequences of red/processed meat consumption. Most of the hypothesized relations between dimensions were therefore supported by data, with message-induced fear positively influencing systematic processing (H1a) and message evaluation (H1b), message-induced anger negatively influencing systematic processing (H2a) and message evaluation (H2b), systematic processing positively influencing message evaluation (H3), positive message evaluation leading to higher attitude toward RPMC reduction (H5), and positive attitude toward reduced RPMC predicting lower intention to eat red/processed meat (H6).

The comparison of the four message conditions on the integrated model allowed us to answer our research questions about the differential impact of the cognitive and emotional dimensions after exposure to differently framed messages. First, message-induced fear increased systematic processing in all conditions, and especially in the loss and gain message conditions (RQ1a). It also positively increased message evaluation in all conditions, especially in the case of non-gain messages (RQ1b).

Second, message-induced anger inhibited systematic processing in all conditions, and especially in the gain condition (RQ2a). It also led to a more negative evaluation of the message, especially in the non-loss condition (RQ2b). Third, systematic processing positively influenced message evaluation in all conditions, and especially in the loss message condition (RQ3), while heuristic processing negatively influenced message evaluation in the non-gain and loss message conditions (RQ4). Fourth, in the case of gain and non-loss messages, but not in the case of non-gain and loss messages, a more positive evaluation of the message (activated by systematic processing) led to higher attitude toward reduced RPMC (RQ5), which in turn led to lower intention to eat red/processed meat (RQ6). In regard to non-gain messages, attitude toward reduced RPMC was positively predicted by message-induced fear and negatively predicted by message-induced anger, showing that such messages influenced attitude mainly through an emotional reaction. Finally, in regard to loss messages, attitude was instead only predicted by baseline attitude.

## Discussion

The results of the present study clarify the emotional and cognitive mechanisms underlying the effects of health messages about reduced RPMC formulated with four different frames: gain, non-loss, non-gain, and loss. While gain messages presented the positive health outcomes deriving from reduced RPMC, non-loss messages informed receivers about the avoidance of negative health consequences through reduced RMPC, non-gain messages provided information about the missed positive health outcomes connected with high RPMC, and finally loss messages focused on the negative health outcomes connected with high RPMC. Using Bayesian analyses and comparing the fit of different models, we found that a model including both cognitive (systematic and heuristic processing of the messages) and emotional dimensions (message-induced fear and message-induced anger) leads to better understanding of how message evaluation predicts receivers' attitude and intention toward red/processed meat consumption, and has a better fit than models considering only either cognitive or emotional dimensions. We also assessed that some of the relationships among dimensions included in the model have different weight or even disappear in different message frames.

The above results offer two main contributions to research on framing effects in communication aimed to reduce RPMC. The first contribution regards the identification of key variables in the explanation of how cognitive and emotional mechanisms predict receivers' attitude and intention after being exposed to persuasive messages aimed at inducing a reduction of RPMC. Our hypothesized model, which was confirmed by the data, showed that message exposure activates a chain of emotional and cognitive reactions which end up influencing receivers' evaluation of the messages and, in turn, subsequent attitude and intention toward RPMC. More specifically, emotional reactions strongly influenced cognitive processing. Fear elicited by the messages was associated with systematic processing of the messages themselves. This in turn led to a positive evaluation of the message and increased positive attitude toward reducing RPMC and a lower intention to eat red/processed meat in the future. Conversely, anger elicited by the messages was associated with heuristic processing of the messages, which did not lead to any change in attitude or intention toward RPMC.

The second main contribution of our research regards the comparative analysis of how the four different message frames activated specific cognitive and emotional mechanisms and, in turn, affected attitude and intention. First, we showed that systematic processing positively influenced message evaluation in all message conditions. In the case of gain and non-loss message conditions, this positive evaluation in turn led to higher attitude toward reduced RPMC and lower intention toward RPMC. These results suggest that presenting the positive consequences (gain) or the avoidance of negative consequences (non-loss) connected with reduced RPMC activates successful systematic processing of the message, which in turn influences message evaluation, attitude, and intention. The observed key role of systematic processing and its effects are consistent with the dual-process models of persuasion (Petty and Cacioppo, 1986; Eagly and Chaiken, 1993). In the case of non-gain and loss messages, systematic process also led to a more positive evaluation of the message, but this effect did not reverberate on a change in attitude or intention toward RPMC. This result suggests that cognitively processing the missed positive consequences (non-gain) or the negative consequences (loss) associated with reduced RPMC interrupted the persuasive effect of the message on receivers' attitude and intention after the message evaluation. Why this interruption occurs might be explained in the light of the role of the emotional factors, as commented below.

We also showed how emotions influenced the systematic processing of the message in the various message conditions. In all conditions the perception of fear activated a fruitful chain of message elaboration (via systematic processing and then a positive evaluation of the message), leading to higher attitude toward reduction and lower intention to eat red/processed meat, albeit only in the case of gain and non-loss messages. These results support the idea of fear as being a compelling persuader (Tomkins, 1984), able to direct cognitive processes (Izard, 1993). As shown by a long history of research, inducing fear is an effective communication strategy to influence receivers' attitude and intention, given its ability to stimulate systematic processing based on a large number of issue-relevant thoughts (e.g., Meijnders et al., 2001; Slater et al., 2002; Meyers-Levy and Maheswaran, 2004; De Hoog et al., 2007). This is more likely to happen when fear is moderate. In our study, gain and non-loss messages very likely stimulated a moderate level of fear, that motivated central processing. However, in the case of gain messages the perception of fear was reduced by participants' positive attitude toward the reduction of their RPMC at T1. This finding may be seen in the light of the cognitive dissonance theory and the related confirmatory bias (Festinger, 1962; Nickerson, 1998). Receivers who positively evaluated reduced meat consumption but ate more meat than recommended, and who received information about the benefits associated with reduced consumption, were possibly confronted with an experience of an inconsistency between their attitude and their behavior. In this case, they might have limited the systematic processing of gain messages to avoid contradictory information (confirmation bias).

Unlike the case of gain messages, in the case of non-loss messages the perception of fear activated a successful chain of message elaboration, regardless of receivers' attitude at T1. Thus, non-loss messages can be considered as the most efficient frame in inducing attitude and intention via a fruitful emotional, and then cognitive message processing that leads to attitude and intention change. This promising effect of the non-loss message can be partially attributed to loss aversion and negativity bias (Kühberger et al., 1999). Proposing the avoidance of negative outcomes directs the attention to the possible negative consequences of one's behavior and triggers some fear. Consequently, the acquisition of fearful and negative information induces greater information processing than does positive information, as suggested by the negativity bias theory (Rozin and Royzman, 2001). A greater elaboration may then induce greater attitude and intention change. This finding is consistent with a prior study by Carfora et al. (2020b), also comparing gain, non-loss, non-gain, and loss messages, and showing that non-loss messages were indeed the most effective messages, apt to involve and persuade the majority of receivers, independent of their prior beliefs.

In the case of loss messages, the elicitation of fear led to systematic processing and a positive evaluation of the message content. Relying on a negative bias, the acquisition of negative information led to greater information processing than the acquisition of positive information. However, the elaboration of loss messages did not converge on higher attitude and lower intention toward red/processed meat consumption, probably because it activated high levels of fear that in turn induced resistance to the message (Witte, 1992; Ruiter et al., 2001). The loss-framed messages were therefore not effective in impacting on attitude and intention at T2. These findings contribute to further clarify when message-induced fear becomes counterproductive (Popova, 2014). In the case of loss messages, fear probably acted as a cue for people to use defensive strategies, to reduce potential emotional distress associated with the read messages (Witte, 1992; Ruiter et al., 2001).

Finally, in the case of non-gain messages a further different effect of induced fear emerged. Fear directly predicted higher attitude toward reduced RPMC and lower intention to eat red/processed meat, regardless of systematic processing of the message. Probably, the non-gain frame scared successfully participants, not activating defensive strategies but also bypassing systematic processing. This latest point may be counterproductive long-term in regard to the persuasiveness of this message frame because, according to the dual-process model, only when receivers activate systematic processing is the message internalized, resulting in a longer and more stable attitude change (Eagly and Chaiken, 1993).

Anger also played a relevant role in influencing systematic processing and evaluation of the messages, but in a negative direction. This result is coherent with previous evidence according to which anger mobilizes cognitive mechanics for the purpose of defending oneself, and these include resistance to a message inducing anger (Brown, 2001). It is also consistent with previous research showing that angry people tend to use heuristics to process information (Moons and Mackie, 2007). Similar to what happened for fear, in the non-gain message condition (and not in the other conditions) anger was directly related to a negative attitude toward reducing RPMC at T2.

Our research has several limitations. First, in the light of the existing gap between intentions to perform a certain behavior and its actual performance (Hagger and Chatzisarantis, 2014), the lack of a measurement of the actual behavior is the most important limitation of the present study. Second, our sample was restricted to Italian people, thus the data may not be generalized to other countries. Third, the measures used in our questionnaire lacked manipulation checks. Fourth, we cannot exclude the risk of self-selection bias, as participants were invited for a study on public communication. Fifth, we did not adopt an open-science approach by pre-registering our hypotheses and analysis plan. Finally, participants were exposed only once to short messages on health outcomes, thus we were able to assess only small and short-term effects. Messages delivered over a longer time span and with repeated exposure (e.g., Caso and Carfora, 2017; Carfora et al., 2018) could yield larger and long-term effects on recipients' attitudes and intentions.

Future research should carefully retest our preliminary results on the mechanisms involved in processing messages on RPMC formulated with different frames, sending messages over a longer period of time. Moreover, future studies could verify whether gain-, non-loss-, non-gain-, and loss-framed messages systematically differ in the level of positive emotions they engender, and whether message-induced positive emotions are linked directly to persuasive outcomes. Future studies could also verify whether the cognitive and emotional processing of gain, non-loss, non-gain, and loss messages are the same when the presented outcomes are different from the ones presented here. We cannot exclude that there might be systematic differences among messages that propose the same behavior to obtain different outcomes. For example, the reduction of RPMC to avoid negative environmental consequences could be felt as too distant in time and thus non-loss frames could resonate as less convincing than was the case in the present study. Similarly, a close consideration of how health messages focused on different recommended behavior (e.g., sugary food and junk food consumption) which may align with one frame type over another would be useful. Future studies could also deepen our understanding of the effects of the four types of message frames considering their fit with individual characteristics, such as the utilitarian or hedonic approach toward food purchasing (Lombardi et al., 2017), or consumers' trust toward the health recommendation provided by public authorities (Carfora et al., 2019c; Cembalo et al., 2019).

## Conclusion

To sum up, in the present study we validated a model explaining how messages differing according to the regulatory framework model (i.e., gain, non-loss, non-gain, and loss messages) influence receivers' evaluation of the messages, as well as attitude and intention toward red/processed meat consumption. Our results respond to the need for theoretical advancement in the area of the underlying mechanisms elicited by message framing (Rothman et al., 2020) and show the plausibility of a model including both the cognitive and emotional dimensions elicited by message exposure. Starting from the assumption that both cognitive and emotional mechanisms underlie the persuasiveness of a message, we showed that a model of persuasion that articulates how message-induced negative emotions may influence information processing and subsequent attitude and intention. In the case of gain and especially non-loss messages, systematic processing, supported by a moderate level of fear, strongly contributed to the persuasive effect of the messages. Instead, the effects of loss and non-gain messages were more determined by emotional reactions and less mediated by systematic processing, ending up with reduced persuasive power.

In conclusion, our study introduced and tested an inclusive reference model to explain the effects of message frames based on the presence/absence of positive/negative outcomes of expected behaviors and aimed at changing the attitudes and behaviors of the receivers. It will be up to future research to further investigate the possibility of applying this model to messages aimed at modifying attitudes and intentions other than the one investigated here, as well as verifying if and how the differences in the mechanisms studied here also depend on individual differences among receivers.

## Data Availability Statement

The raw data supporting the conclusions of this article will be made available by the authors, without undue reservation.

## Ethics Statement

The studies involving human participants were reviewed and approved by Ethics Committee of the Department of Psychology—CoCatholic University of the Sacred Heart. The patients/participants provided their written informed consent to participate in this study.

## Author Contributions

VC proposed the research questions, planned the research design, and took responsibility for data collection and the manuscript. MP analyzed the data and supervised their interpretation. PC supervised conception, research design, and interpretation of data. She also thoroughly revised the manuscript in regard to content and style. All authors contributed to the article and approved the submitted version.

## Conflict of Interest

The authors declare that the research was conducted in the absence of any commercial or financial relationships that could be construed as a potential conflict of interest.
