# Supplementary material for: A Cognitive-Emotional Model to Explain Message Framing Effects: Reducing Meat Consumption
Source: Front Psychol. 2021 Mar 29;12:583209. doi: 10.3389/fpsyg.2021.583209 (PMC8039126; doi:10.3389/fpsyg.2021.583209)
Supplement: Supplementary file 1 [file Table_1.DOCX]

**Appendix 1**

*Messages delivered in the four Message Conditions.*

| **Gain Messages** | **Non-Loss Messages** | **Non-Gain Messages** | **Loss Messages** |
| --- | --- | --- | --- |
| If you eat little red meat and cold cuts, *you will improve* the health of your stomach. | If you eat little red meat and cold cuts, *you will avoid damaging* the health of your stomach. | If you eat much red meat and cold cuts, *you will miss the chance to improve* the health of your stomach. | If you eat much red meat and cold cuts, *you will damage* the health of your stomach. |
| If you eat little red meat and cold cuts, *you will improve* the functioning of your bowel. | If you eat little red meat and cold cuts, *you will avoid damaging* the functioning of your bowel. | If you eat little red meat and cold cuts, *you will miss the opportunity to improve* the functioning of your bowel. | If you eat much red meat and cold cuts, *you will damage* the functioning of your bowel. |
| If you eat little red meat and cold cuts, *you will improve* the functionality of your heart. | If you eat little red meat and cold cuts, *you will avoid worsening* the functionality of your heart. | If you eat little red meat and cold cuts, *you will miss the chance to improve* the functionality of your heart. | If you eat much red meat and cold cuts, *you will worsen* the functionality of your heart. |
| If you eat little red meat and cold cuts, *you will improve the proper functioning* of your arteries. | If you eat little red meat and cold cuts, *you will avoid worsening* the functioning of your arteries. | If you eat little red meat and cold cuts, *you will miss the opportunity to improve* the proper functioning of your arteries. | If you eat much red meat and cold cuts, *you will worsen* the functioning of your arteries. |
| If you eat little red meat and cold cuts, *you will enhance* the functionality of your kidneys. | If you eat little red meat and cold cuts, *you will avoid straining* the functionality of your kidneys. | If you eat little red meat and cold cuts, *you will miss the chance to enhance* the functionality of your kidneys. | If you eat much red meat and cold cuts, *you will strain* the functionality of your kidneys. |
| If you eat little red meat and cold cuts, *you will enhance* the health of your lungs. | If you eat little red meat and cold cuts, *you will avoid damaging* the health of your lungs. | If you eat little red meat and cold cuts, *you will miss the chance to enhance* the health of your lungs. | If you eat much red meat and cold cuts, *you will damage* your lungs. |
| If you eat little red meat and cold cuts, *you will enhance* the health of your pancreas. | If you eat little red meat and cold cuts, *you will avoid damaging* the health of your pancreas. | If you eat little red meat and cold cuts, *you will miss the chance to enhance* the health of your pancreas. | If you eat much red meat and cold cuts, *you will damage* your pancreas. |
| If you eat little red meat and cold cuts, *you will improve* the chance of having an optimal blood pressure. | If you eat little red meat and cold cuts, *you will decrease the chance* of having hypertension. | If you eat little red meat and cold cuts, *you will miss the chance* of having an optimal blood pressure. | If you eat much red meat and cold cuts, *you will increase* the chance of having hypertension. |
