# Supplementary material for: A Cognitive-Emotional Model to Explain Message Framing Effects: Reducing Meat Consumption
Source: Front Psychol. 2021 Mar 29;12:583209. doi: 10.3389/fpsyg.2021.583209 (PMC8039126; doi:10.3389/fpsyg.2021.583209)
Supplement: Supplementary file 2 [file Table_2.DOCX]

## Appendix 2

## *Parameters Details of Model 4*

The following boxes show the estimates of the model parameters in the four groups (gain, non-loss, non-gain, loss) divided by type of parameter: intercepts (box 1), regression coefficients (box 2 - 5), and residual variances (box 6).

**Box 1**

*Intercepts of all Message Conditions*

| **Gain Message Condition** | | | | | | |
| --- | --- | --- | --- | --- | --- | --- |
|  | *Estimate* | *Post.SD* | *Pi.lower* | *Pi.upper* | *Rhat* | *Prior* |
| Message Induced Fear at T2 | 1.80 | 0.267 | 1.28 | 2.32 | 1.00 | normal(4,1) |
| Message Induced Anger at T2 | 2.22 | 0.30 | 1.63 | 2.80 | 1.00 | normal(4,1) |
| Systematic Processing at T2 | 3.80 | 0.52 | 2.80 | 4.81 | 1.00 | normal(4,1) |
| Heuristic Processing at T2 | 2.50 | 0.52 | 1.50 | 3.53 | 1.00 | normal(4,1) |
| Message Evaluation at T2 | 2.14 | 0.53 | 1.11 | 3.22 | 1.00 | normal(4,1) |
| Attitude at T2 | 3.78 | 0.57 | 2.67 | 4.89 | 1.00 | normal(4,1) |
| Intention at T2 | 3.72 | 0.48 | 2.80 | 4.66 | 1.00 | normal(4,1) |
| **Non-loss Message Condition** | | | | | | |
| Message Induced Fear at T2 | 2.11 | 0.35 | 1.41 | 2.80 | 1.00 | normal(4,1) |
| Message Induced Anger at T2 | 2.15 | 0.37 | 1.44 | 2.84 | 1.00 | normal(4,1) |
| Systematic Processing at T2 | 4.66 | 0.50 | 3.69 | 5.64 | 1.00 | normal(4,1) |
| Heuristic Processing at T2 | 3.52 | 0.52 | 2.50 | 4.56 | 1.00 | normal(4,1) |
| Message Evaluation at T2 | 2.94 | 0.62 | 1.72 | 4.17 | 1.00 | normal(4,1) |
| Attitude at T2 | 2.51 | 0.67 | 1.23 | 3.84 | 1.00 | normal(4,1) |
| Intention at T2 | 3.84 | 0.57 | 2.71 | 4.96 | 1.00 | normal(4,1) |
| **Non-gain Message Condition** | | | | | | |
| Message Induced Fear at T2 | 2.31 | 0.42 | 1.50 | 3.13 | 1.00 | normal(4,1) |
| Message Induced Anger at T2 | 1.96 | 0.45 | 1.11 | 2.84 | 1.00 | normal(4,1) |
| Systematic Processing at T2 | 4.02 | 0.65 | 2.76 | 5.27 | 1.00 | normal(4,1) |
| Heuristic Processing at T2 | 3.24 | 0.64 | 2.00 | 4.49 | 1.00 | normal(4,1) |
| Message Evaluation at T2 | 2.05 | 0.64 | 0.82 | 3.29 | 1.00 | normal(4,1) |
| Attitude at T2 | 4.85 | 0.65 | 3.57 | 6.12 | 1.00 | normal(4,1) |
| Intention at T2 | 3.33 | 0.54 | 2.26 | 4.38 | 1.00 | normal(4,1) |
| **Loss Message Condition** | | | | | | |
| Message Induced Fear at T2 | 2.55 | 0.37 | 1.84 | 3.27 | 1.00 | normal(4,1) |
| Message Induced Anger at T2 | 2.56 | 0.41 | 1.77 | 3.37 | 1.00 | normal(4,1) |
| Systematic Processing at T2 | 3.36 | 0.59 | 2.20 | 4.49 | 1.00 | normal(4,1) |
| Heuristic Processing at T2 | 3.66 | 0.49 | 2.70 | 4.64 | 1.00 | normal(4,1) |
| Message Evaluation at T2 | 1.47 | 0.56 | 0.38 | 2.60 | 1.00 | normal(4,1) |
| Attitude at T2 | 3.54 | 0.66 | 2.26 | 4.80 | 1.00 | normal(4,1) |
| Intention at T2 | 2.89 | 0.51 | 1.89 | 3.89 | 1.00 | normal(4,1) |

**Box 2**

*Regression Parameters of the Gain Message Condition*

| **Gain Message Condition** | | | | | | |
| --- | --- | --- | --- | --- | --- | --- |
|  | *Estimate* | *Post.SD* | *Pi.lower* | *Pi.upper* | *Rhat* | *Prior* |
| **Message Induced Fear at T2** |  |  |  |  |  |  |
| Attitude at T1 | -0.07 | 0.03 | -0.14 | -0.00 | 1.00 | normal(0,2) |
| Intention at T1 | 0.02 | 0.05 | -0.07 | 0.11 | 1.00 | normal(0,2) |
|  |  |  |  |  |  |  |
| **Message Induced Anger at T2** |  |  |  |  |  |  |
| Attitude at T1 | -0.17 | 0.04 | -0.25 | -0.10 | 1.00 | normal(0,2) |
| Intention at T1 | 0.05 | 0.05 | -0.05 | 0.16 | 1.00 | normal(0,2) |
|  |  |  |  |  |  |  |
| **Systematic Processing at T2** |  |  |  |  |  |  |
| Attitude at T1 | 0.13 | 0.06 | 0.00 | 0.26 | 1.00 | normal(0,2) |
| Intention at T1 | 0.05 | 0.09 | -0.12 | 0.22 | 1.00 | normal(0,2) |
| Message Induced Fear at T2 | 0.80 | 0.17 | 0.46 | 1.14 | 1.00 | normal(0,2) |
| Message Induced Anger at T2 | -0.92 | 0.15 | -1.22 | -0.63 | 1.00 | normal(0,2) |
|  |  |  |  |  |  |  |
| **Heuristic Processing at T2** |  |  |  |  |  |  |
| Attitude at T1 | -0.02 | 0.06 | -0.15 | 0.10 | 1.00 | normal(0,2) |
| Intention at T1 | 0.09 | 0.08 | -0.07 | 0.26 | 1.00 | normal(0,2) |
| Message Induced Fear at T2 | -0.25 | 0.17 | -0.58 | 0.07 | 1.00 | normal(0,2) |
| Message Induced Anger at T2 | 0.45 | 0.15 | 0.17 | 0.73 | 1.00 | normal(0,2) |
|  |  |  |  |  |  |  |
| **Message Evaluation at T2** |  |  |  |  |  |  |
| Attitude at T1 | -0.01 | 0.06 | -0.12 | 0.11 | 1.00 | normal(0,2) |
| Intention at T1 | -0.08 | 0.07 | -0.22 | 0.06 | 1.00 | normal(0,2) |
| Message Induced Fear at T2 | 0.40 | 0.15 | 0.10 | 0.70 | 1.00 | normal(0,2) |
| Message Induced Anger at T2 | -0.55 | 0.14 | -0.83 | -0.28 | 1.00 | normal(0,2) |
| Systematic Processing at T2 | 0.62 | 0.07 | 0.48 | 0.76 | 1.00 | normal(0,2) |
| Heuristic Processing at T2 | -0.06 | 0.07 | -0.21 | 0.09 | 1.00 | normal(0,2) |
|  |  |  |  |  |  |  |
| **Attitude at T2** |  |  |  |  |  |  |
| Attitude at T1 | 0.09 | 0.06 | -0.03 | 0.22 | 1.00 | normal(0,2) |
| Intention at T1 | -0.30 | 0.08 | -0.46 | -0.14 | 1.00 | normal(0,2) |
| Message Induced Fear at T2 | -0.31 | 0.17 | -0.65 | 0.03 | 1.00 | normal(0,2) |
| Message Induced Anger at T2 | 0.16 | 0.16 | -0.15 | 0.48 | 1.00 | normal(0,2) |
| Systematic Processing at T2 | 0.19 | 0.11 | -0.02 | 0.39 | 1.00 | normal(0,2) |
| Heuristic Processing at T2 | -0.01 | 0.08 | -0.17 | 0.15 | 1.00 | normal(0,2) |
| Message Evaluation at T2 | 0.33 | 0.10 | 0.13 | 0.53 | 1.00 | normal(0,2) |
|  |  |  |  |  |  |  |
| **Intention at T2** |  |  |  |  |  |  |
| Attitude at T1 | 0.02 | 0.05 | -0.08 | 0.11 | 1.00 | normal(0,2) |
| Intention at T1 | 0.40 | 0.06 | 0.28 | 0.52 | 1.00 | normal(0,2) |
| Message Induced Fear at T2 | -0.36 | 0.13 | -0.61 | -0.11 | 1.00 | normal(0,2) |
| Message Induced Anger at T2 | 0.16 | 0.12 | -0.07 | 0.40 | 1.00 | normal(0,2) |
| Systematic Processing at T2 | 0.11 | 0.08 | -0.04 | 0.27 | 1.00 | normal(0,2) |
| Heuristic Processing at T2 | -0.01 | 0.06 | -0.13 | 0.10 | 1.00 | normal(0,2) |
| Message Evaluation at T2 | -0.07 | 0.08 | -0.22 | 0.07 | 1.00 | normal(0,2) |
| Attitude at T2 | -0.22 | 0.06 | -0.34 | -0.09 | 1.00 | normal(0,2) |

**Box 3**

*Regression Parameters of the Non-Loss Message Condition*

| **Non-Loss Message Condition** | | | | | | |
| --- | --- | --- | --- | --- | --- | --- |
|  | *Estimate* | *Post.SD* | *Pi.lower* | *Pi.upper* | *Rhat* | *Prior* |
| **Message Induced Fear at T2** |  |  |  |  |  |  |
| Attitude at T1 | -0.04 | 0.04 | -0.12 | 0.05 | 1.000 | normal(0,2) |
| Intention at T1 | -0.061 | 0.06 | -0.18 | 0.05 | 1.000 | normal(0,2) |
|  |  |  |  |  |  |  |
| **Message Induced Anger at T2** |  |  |  |  |  |  |
| Attitude at T1 | -0.13 | 0.05 | -0.22 | -0.03 | 1.000 | normal(0,2) |
| Intention at T1 | 0.02 | 0.06 | -0.10 | 0.15 | 1.000 | normal(0,2) |
|  |  |  |  |  |  |  |
| **Systematic Processing at T2** |  |  |  |  |  |  |
| Attitude at T1 | 0.14 | 0.06 | 0.02 | 0.26 | 1.000 | normal(0,2) |
| Intention at T1 | -0.15 | 0.08 | -0.30 | 0.01 | 1.000 | normal(0,2) |
| Message Induced Fear at T2 | 0.61 | 0.14 | 0.33 | 0.90 | 1.000 | normal(0,2) |
| Message Induced Anger at T2 | -0.64 | 0.14 | -0.91 | -0.38 | 1.000 | normal(0,2) |
|  |  |  |  |  |  |  |
| **Heuristic Processing at T2** |  |  |  |  |  |  |
| Attitude at T1 | -0.08 | 0.06 | -0.21 | 0.04 | 1.000 | normal(0,2) |
| Intention at T1 | -0.13 | 0.09 | -0.30 | 0.04 | 1.000 | normal(0,2) |
| Message Induced Fear at T2 | -0.15 | 0.15 | -0.44 | 0.14 | 1.000 | normal(0,2) |
| Message Induced Anger at T2 | -0.13 | 0.09 | -0.30 | 0.04 | 1.000 | normal(0,2) |
|  |  |  |  |  |  |  |
| **Message Evaluation at T2** |  |  |  |  |  |  |
| Attitude at T1 | -0.00 | 0.06 | -0.13 | 0.12 | 1.000 | normal(0,2) |
| Intention at T1 | -0.16 | 0.08 | -0.31 | -0.00 | 1.000 | normal(0,2) |
| Message Induced Fear at T2 | 0.46 | 0.15 | 0.17 | 0.75 | 1.000 | normal(0,2) |
| Message Induced Anger at T2 | -0.62 | 0.14 | -0.91 | -0.35 | 1.000 | normal(0,2) |
| Systematic Processing at T2 | 0.55 | 0.08 | 0.40 | 0.71 | 1.000 | normal(0,2) |
| Heuristic Processing at T2 | -0.09 | 0.08 | -0.25 | 0.06 | 1.000 | normal(0,2) |
|  |  |  |  |  |  |  |
| **Attitude at T2** |  |  |  |  |  |  |
| Attitude at T1 | 0.43 | 0.06 | 0.30 | 0.55 | 1.000 | normal(0,2) |
| Intention at T1 | -0.15 | 0.09 | -0.32 | 0.02 | 1.000 | normal(0,2) |
| Message Induced Fear at T2 | -0.03 | 0.17 | -0.35 | 0.30 | 1.000 | normal(0,2) |
| Message Induced Anger at T2 | 0.00 | 0.16 | -0.33 | 0.32 | 1.000 | normal(0,2) |
| Systematic Processing at T2 | -0.06 | 0.11 | -0.27 | 0.16 | 1.000 | normal(0,2) |
| Heuristic Processing at T2 | -0.02 | 0.09 | -0.19 | 0.15 | 1.000 | normal(0,2) |
| Message Evaluation at T2 | 0.34 | 0.10 | 0.15 | 0.53 | 1.000 | normal(0,2) |
|  |  |  |  |  |  |  |
| **Intention at T2** |  |  |  |  |  |  |
| Attitude at T1 | 0.01 | 0.06 | -0.11 | 0.13 | 1.00 | normal(0,2) |
| Intention at T1 | 0.44 | 0.7 | 0.30 | 0.58 | 1.00 | normal(0,2) |
| Message Induced Fear at T2 | -0.13 | 0.13 | -0.39 | 0.13 | 1.00 | normal(0,2) |
| Message Induced Anger at T2 | -0.02 | 0.13 | -0.28 | 0.23 | 1.00 | normal(0,2) |
| Systematic Processing at T2 | 0.04 | 0.09 | -0.13 | 0.21 | 1.00 | normal(0,2) |
| Heuristic Processing at T2 | -0.10 | 0.07 | -0.24 | 0.03 | 1.00 | normal(0,2) |
| Message Evaluation at T2 | -0.06 | 0.08 | -0.22 | 0.10 | 1.00 | normal(0,2) |
| Attitude at T2 | -0.16 | 0.07 | -0.30 | -0.02 | 1.00 | normal(0,2) |

**Box 4**

*Regression Parameters of the Non-Gain Message Condition*

| **Non-Gain Message Condition** | | | | | | |
| --- | --- | --- | --- | --- | --- | --- |
|  | *Estimate* | *Post.SD* | *Pi.lower* | *Pi.upper* | *Rhat* | *Prior* |
| **Message Induced Fear at T2** |  |  |  |  |  |  |
| Attitude at T1 | -0.06 | 0.05 | -0.16 | 0.04 | 1.00 | normal(0,2) |
| Intention at T1 | -0.08 | 0.06 | -0.21 | 0.04 | 1.00 | normal(0,2) |
|  |  |  |  |  |  |  |
| **Message Induced Anger at T2** |  |  |  |  |  |  |
| Attitude at T1 | -0.05 | 0.05 | -0.16 | 0.06 | 1.00 | normal(0,2) |
| Intention at T1 | 0.01 | 0.07 | -0.13 | 0.15 | 1.00 | normal(0,2) |
|  |  |  |  |  |  |  |
| **Systematic Processing at T2** |  |  |  |  |  |  |
| Attitude at T1 | 0.09 | 0.08 | -0.07 | 0.24 | 1.00 | normal(0,2) |
| Intention at T1 | -0.12 | 0.10 | -0.32 | 0.09 | 1.00 | normal(0,2) |
| Message Induced Fear at T2 | 0.67 | 0.15 | 0.37 | 0.98 | 1.00 | normal(0,2) |
| Message Induced Anger at T2 | -0.45 | 0.15 | -0.74 | -0.15 | 1.00 | normal(0,2) |
|  |  |  |  |  |  |  |
| **Heuristic Processing at T2** |  |  |  |  |  |  |
| Attitude at T1 | -0.10 | 0.08 | -0.25 | 0.05 | 1.00 | normal(0,2) |
| Intention at T1 | 0.01 | 0.10 | -0.19 | 0.21 | 1.00 | normal(0,2) |
| Message Induced Fear at T2 | 0.19 | 0.15 | -0.19 | 0.40 | 1.00 | normal(0,2) |
| Message Induced Anger at T2 | 0.21 | 0.15 | -0.08 | 0.49 | 1.00 | normal(0,2) |
|  |  |  |  |  |  |  |
| **Message Evaluation at T2** |  |  |  |  |  |  |
| Attitude at T1 | 0.00 | 0.07 | -0.14 | 0.14 | 1.00 | normal(0,2) |
| Intention at T1 | -0.10 | 0.09 | -0.28 | 0.07 | 1.00 | normal(0,2) |
| Message Induced Fear at T2 | 0.79 | 0.14 | 0.51 | 1.07 | 1.00 | normal(0,2) |
| Message Induced Anger at T2 | -0.52 | 0.13 | -0.78 | -0.25 | 1.00 | normal(0,2) |
| Systematic Processing at T2 | 0.58 | 0.07 | 0.44 | 0.72 | 1.00 | normal(0,2) |
| Heuristic Processing at T2 | -0.19 | 0.08 | -0.34 | -0.04 | 1.00 | normal(0,2) |
|  |  |  |  |  |  |  |
| **Attitude at T2** |  |  |  |  |  |  |
| Attitude at T1 | 0.17 | 0.07 | 0.03 | 0.31 | 1.00 | normal(0,2) |
| Intention at T1 | -0.36 | 0.09 | -0.54 | -0.18 | 1.00 | normal(0,2) |
| Message Induced Fear at T2 | 0.33 | 0.17 | 0.00 | 0.66 | 1.00 | normal(0,2) |
| Message Induced Anger at T2 | -0.43 | 0.14 | -0.71 | -0.15 | 1.00 | normal(0,2) |
| Systematic Processing at T2 | 0.07 | 0.10 | -0.13 | 0.26 | 1.00 | normal(0,2) |
| Heuristic Processing at T2 | 0.08 | 0.08 | -0.07 | 0.24 | 1.00 | normal(0,2) |
| Message Evaluation at T2 | 0.11 | 0.09 | -0.07 | 0.29 | 1.00 | normal(0,2) |
|  |  |  |  |  |  |  |
| **Intention at T2** |  |  |  |  |  |  |
| Attitude at T1 | -0.00 | 0.05 | -0.10 | 0.1 | 1.00 | normal(0,2) |
| Intention at T1 | 0.38 | 0.07 | 0.25 | 0.51 | 1.00 | normal(0,2) |
| Message Induced Fear at T2 | -0.24 | 0.11 | -0.46 | -0.02 | 1.00 | normal(0,2) |
| Message Induced Anger at T2 | -0.02 | 0.010 | -0.21 | 0.18 | 1.00 | normal(0,2) |
| Systematic Processing at T2 | 0.10 | 0.07 | -0.03 | 0.23 | 1.00 | normal(0,2) |
| Heuristic Processing at T2 | 0.05 | 0.05 | -0.06 | 0.16 | 1.00 | normal(0,2) |
| Message Evaluation at T2 | -0.06 | 0.06 | -0.18 | 0.07 | 1.00 | normal(0,2) |
| Attitude at T2 | -0.12 | 0.06 | -0.23 | -0.00 | 1.00 | normal(0,2) |

**Box 5**

*Regression Parameters of the Loss Message Condition*

| **Loss Message Condition** | | | | | | |
| --- | --- | --- | --- | --- | --- | --- |
|  | *Estimate* | *Post.SD* | *Pi.lower* | *Pi.upper* | *Rhat* | *Prior* |
| **Message Induced Fear at T2** |  |  |  |  |  |  |
| Attitude at T1 | -0.08 | 0.04 | -0.17 | 0.00 | 1.00 | normal(0,2) |
| Intention at T1 | -0.11 | 0.06 | -0.23 | 0.01 | 1.00 | normal(0,2) |
|  |  |  |  |  |  |  |
| **Message Induced Anger at T2** |  |  |  |  |  |  |
| Attitude at T1 | -0.13 | 0.05 | -0.23 | -0.03 | 1.00 | normal(0,2) |
| Intention at T1 | -0.05 | 0.07 | -0.18 | 0.09 | 1.00 | normal(0,2) |
|  |  |  |  |  |  |  |
| **Systematic Processing at T2** |  |  |  |  |  |  |
| Attitude at T1 | 0.08 | 0.07 | -0.05 | 0.21 | 1.00 | normal(0,2) |
| Intention at T1 | 0.12 | 0.09 | -0.06 | 0.30 | 1.00 | normal(0,2) |
| Message Induced Fear at T2 | 0.91 | 0.15 | 0.62 | 1.20 | 1.00 | normal(0,2) |
| Message Induced Anger at T2 | -0.79 | 0.13 | -1.05 | -0.52 | 1.00 | normal(0,2) |
|  |  |  |  |  |  |  |
| **Heuristic Processing at T2** |  |  |  |  |  |  |
| Attitude at T1 | -0.14 | 0.06 | -0.25 | -0.03 | 1.00 | normal(0,2) |
| Intention at T1 | 0.12 | 0.09 | -0.06 | 0.30 | 1.00 | normal(0,2) |
| Message Induced Fear at T2 | -0.14 | 0.12 | -0.38 | 0.10 | 1.00 | normal(0,2) |
| Message Induced Anger at T2 | 0.23 | 0.11 | 0.02 | 0.45 | 1.00 | normal(0,2) |
|  |  |  |  |  |  |  |
| **Message Evaluation at T2** |  |  |  |  |  |  |
| Attitude at T1 | 0.02 | 0.05 | -0.09 | 0.12 | 1.00 | normal(0,2) |
| Intention at T1 | -0.03 | 0.07 | -0.18 | 0.11 | 1.00 | normal(0,2) |
| Message Induced Fear at T2 | 0.32 | 0.13 | 0.07 | 0.58 | 1.00 | normal(0,2) |
| Message Induced Anger at T2 | -0.30 | 0.11 | -0.53 | -0.08 | 1.00 | normal(0,2) |
| Systematic Processing at T2 | 0.70 | 0.06 | 0.57 | 0.83 | 1.00 | normal(0,2) |
| Heuristic Processing at T2 | -0.15 | 0.08 | -0.31 | -0.00 | 1.00 | normal(0,2) |
|  |  |  |  |  |  |  |
| **Attitude at T2** |  |  |  |  |  |  |
| Attitude at T1 | 0.29 | 0.07 | 0.15 | 0.42 | 1.00 | normal(0,2) |
| Intention at T1 | -0.14 | 0.10 | -0.32 | 0.05 | 1.00 | normal(0,2) |
| Message Induced Fear at T2 | -0.21 | 0.17 | -0.56 | 0.13 | 1.00 | normal(0,2) |
| Message Induced Anger at T2 | 0.00 | 0.15 | -0.30 | 0.30 | 1.00 | normal(0,2) |
| Systematic Processing at T2 | 0.02 | 0.12 | -0.23 | 0.26 | 1.00 | normal(0,2) |
| Heuristic Processing at T2 | -0.04 | 0.10 | -0.24 | 0.17 | 1.00 | normal(0,2) |
| Message Evaluation at T2 | 0.18 | 0.12 | -0.05 | 0.41 | 1.00 | normal(0,2) |
|  |  |  |  |  |  |  |
| **Intention at T2** |  |  |  |  |  |  |
| Attitude at T1 | 0.01 | 0.05 | -0.09 | 0.10 | 1.00 | normal(0,2) |
| Intention at T1 | 0.55 | 0.06 | 0.43 | 0.67 | 1.00 | normal(0,2) |
| Message Induced Fear at T2 | 0.02 | 0.11 | -0.20 | 0.23 | 1.00 | normal(0,2) |
| Message Induced Anger at T2 | -0.03 | 0.10 | -0.22 | 0.16 | 1.00 | normal(0,2) |
| Systematic Processing at T2 | 0.02 | 0.08 | -0.14 | 0.17 | 1.00 | normal(0,2) |
| Heuristic Processing at T2 | -0.03 | 0.07 | -0.16 | 0.10 | 1.00 | normal(0,2) |
| Message Evaluation at T2 | 0.01 | 0.05 | -0.09 | 0.10 | 1.00 | normal(0,2) |
| Attitude at T2 | 0.55 | 0.06 | 0.43 | 0.67 | 1.00 | normal(0,2) |

**Box 6**

*Residual Variance of all Message Conditions*

| **Gain Message Condition** | | | | | | |
| --- | --- | --- | --- | --- | --- | --- |
|  | *Estimate* | *Post.SD* | *Pi.lower* | *Pi.upper* | *Rhat* | *Prior* |
| Message Induced Fear at T2 | 0.36 | 0.04 | 0.28 | 0.46 | 1.00 | gamma(1,.5)[sd] |
| Message Induced Anger at T2 | 0.46 | 0.06 | 0.36 | 0.58 | 1.00 | gamma(1,.5)[sd] |
| Systematic Processing at T2 | 1.29 | 0.17 | 1.00 | 1.67 | 1.00 | gamma(1,.5)[sd] |
| Heuristic Processing at T2 | 1.21 | 0.16 | 0.94 | 1.55 | 1.00 | gamma(1,.5)[sd] |
| Message Evaluation at T2 | 0.83 | 0.11 | 0.65 | 1.07 | 1.00 | gamma(1,.5)[sd] |
| Attitude at T2 | 1.03 | 0.13 | 0.80 | 1.32 | 1.00 | gamma(1,.5)[sd] |
| Intention at T2 | 0.55 | 0.07 | 0.43 | 0.71 | 1.00 | gamma(1,.5)[sd] |
| **Non-Loss Message Condition** | | | | | | |
| Message Induced Fear at T2 | 0.49 | 0.06 | 0.39 | 0.63 | 1.00 | gamma(1,.5)[sd] |
| Message Induced Anger at T2 | 0.55 | 0.07 | 0.43 | 0.70 | 1.00 | gamma(1,.5)[sd] |
| Systematic Processing at T2 | 0.91 | 0.11 | 0.72 | 1.16 | 1.00 | gamma(1,.5)[sd] |
| Heuristic Processing at T2 | 0.99 | 0.12 | 0.78 | 1.27 | 1.00 | gamma(1,.5)[sd] |
| Message Evaluation at T2 | 0.85 | 0.11 | 0.67 | 1.08 | 1.00 | gamma(1,.5)[sd] |
| Attitude at T2 | 1.03 | 0.13 | 0.81 | 1.32 | 1.00 | gamma(1,.5)[sd] |
| Intention at T2 | 0.63 | 0.08 | 0.49 | 0.80 | 1.00 | gamma(1,.5)[sd] |
| **Non-Gain Message Condition** | | | | | | |
| Message Induced Fear at T2 | 0.54 | 0.07 | 0.43 | 0.70 | 1.00 | gamma(1,.5)[sd] |
| Message Induced Anger at T2 | 0.60 | 0.08 | 0.47 | 0.77 | 1.00 | gamma(1,.5)[sd] |
| Systematic Processing at T2 | 1.48 | 0.19 | 1.16 | 1.89 | 1.00 | gamma(1,.5)[sd] |
| Heuristic Processing at T2 | 1.41 | 0.18 | 1.10 | 1.79 | 1.00 | gamma(1,.5)[sd] |
| Message Evaluation at T2 | 1.06 | 0.13 | 0.83 | 1.36 | 1.00 | gamma(1,.5)[sd] |
| Attitude at T2 | 1.14 | 0.15 | 0.89 | 1.46 | 1.00 | gamma(1,.5)[sd] |
| Intention at T2 | 0.52 | 0.07 | 0.40 | 0.66 | 1.00 | gamma(1,.5)[sd] |
| **Loss Message Condition** | | | | | | |
| Message Induced Fear at T2 | 0.48 | 0.06 | 0.38 | 0.60 | 1.00 | gamma(1,.5)[sd] |
| Message Induced Anger at T2 | 0.61 | 0.07 | 0.48 | 0.77 | 1.00 | gamma(1,.5)[sd] |
| Systematic Processing at T2 | 1.17 | 0.14 | 0.93 | 1.47 | 1.00 | gamma(1,.5)[sd] |
| Heuristic Processing at T2 | 0.78 | 0.09 | 0.61 | 0.99 | 1.00 | gamma(1,.5)[sd] |
| Message Evaluation at T2 | 0.66 | 0.08 | 0.51 | 0.84 | 1.00 | gamma(1,.5)[sd] |
| Attitude at T2 | 1.20 | 0.15 | 0.93 | 1.52 | 1.00 | gamma(1,.5)[sd] |
| Intention at T2 | 0.47 | 0.06 | 0.37 | 0.60 | 1.00 | gamma(1,.5)[sd] |
